# Validated graft-specific biomarkers identify patients at risk for chronic graft-versus-host disease and death

**DOI:** 10.1172/JCI168575

**Published:** 2023-08-01

**Authors:** Brent R. Logan, Denggang Fu, Alan Howard, Mingwei Fei, Jianqun Kou, Morgan R. Little, Djamilatou Adom, Fathima A. Mohamed, Bruce R. Blazar, Philip R. Gafken, Sophie Paczesny

**Affiliations:** 1 Division of Biostatistics and Center for International Blood and Marrow Transplant Research, Medical College of Wisconsin, Milwaukee, Wisconsin, USA.; 2 Department of Microbiology and Immunology, Medical University of South Carolina, Charleston, South Carolina, USA.; 3 Be The Match and Center for International Blood and Marrow Transplant Research, Minneapolis, Minnesota, USA.; 4 Department of Pediatrics, Indiana University School of Medicine, Indianapolis, Indiana, USA.; 5 Department of Pediatrics, Division of Blood & Marrow Transplant & Cellular Therapy, University of Minnesota Medical School, Minneapolis, Minnesota, USA.; 6 Proteomics & Metabolomics shared resource, Fred Hutchinson Cancer Research Center, Seattle, Washington, USA.

**Keywords:** Transplantation, Bone marrow transplantation

## Abstract

**BACKGROUND:**

Chronic graft-versus-host disease (cGVHD) is a serious complication of allogeneic hematopoietic cell transplantation (HCT). More accurate information regarding the risk of developing cGVHD is required. Bone marrow (BM) grafts contribute to lower cGVHD, which creates a dispute over whether risk biomarker scores should be used for peripheral blood (PB) and BM.

**METHODS:**

Day 90 plasma proteomics from PB and BM recipients developing cGVHD revealed 5 risk markers that were added to 8 previous cGVHD markers to screen 982 HCT samples of 2 multicenter Blood and Marrow Transplant Clinical Trials Network (BMTCTN) cohorts. Each marker was tested for its association with cause-specific hazard ratios (HRs) of cGVHD using Cox-proportional-hazards models. We paired these clinical studies with biomarker measurements in a mouse model of cGVHD.

**RESULTS:**

Spearman correlations between DKK3 and MMP3 were significant in both cohorts. In BMTCTN 0201 multivariate analyses, PB recipients with 1-log increase in CXCL9 and DKK3 were 1.3 times (95% CI: 1.1–1.4, *P* = 0.001) and 1.9 times (95%CI: 1.1–3.2, *P* = 0.019) and BM recipients with 1-log increase in CXCL10 and MMP3 were 1.3 times (95%CI: 1.0–1.6, *P* = 0.018 and *P* = 0.023) more likely to develop cGVHD. In BMTCTN 1202, PB patients with high CXCL9 and MMP3 were 1.1 times (95%CI: 1.0–1.2, *P* = 0.037) and 1.2 times (95%CI: 1.0–1.3, *P* = 0.009) more likely to develop cGVHD. PB patients with high biomarkers had increased likelihood to develop cGVHD in both cohorts (22%–32% versus 8%–12%, *P* = 0.002 and *P* < 0.001, respectively). Mice showed elevated circulating biomarkers before the signs of cGVHD.

**CONCLUSION:**

Biomarker levels at 3 months after HCT identify patients at risk for cGVHD occurrence.

**FUNDING:**

NIH grants R01CA168814, R21HL139934, P01CA158505, T32AI007313, and R01CA264921.

## Graphical abstract

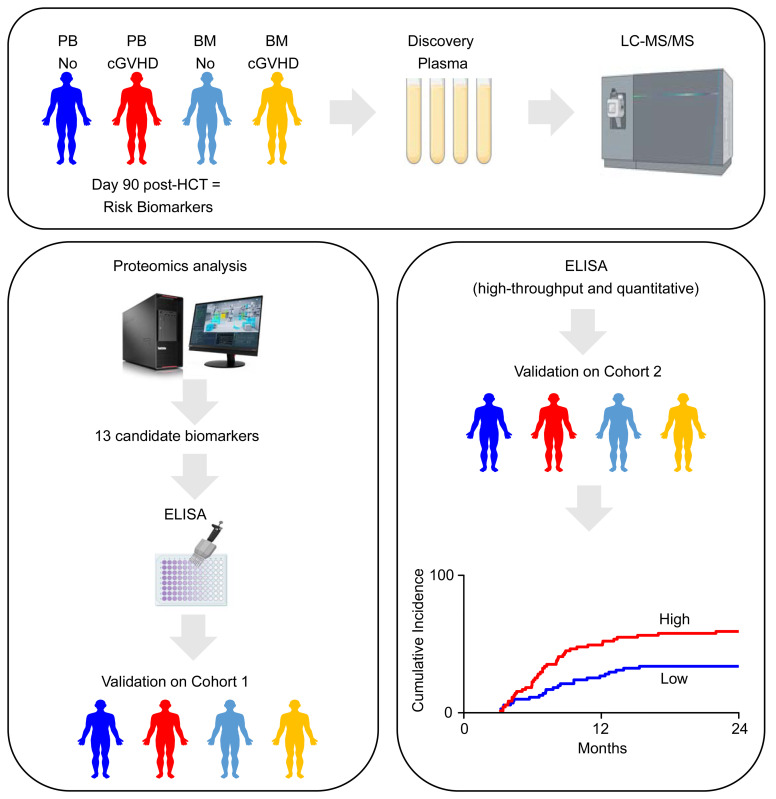

## Introduction

Current estimates of annual numbers of allogeneic hematopoietic cell transplants are approximately 50,000 worldwide, with growth at a rate of 10%–15% per year. Allogeneic hematopoietic cell transplantation (HCT) is a potentially curative therapy for blood cancers and inherited diseases. However, its efficacy has been impeded by chronic graft-versus-host disease (cGVHD) which remains the most common complication in patients surviving more than 90 days and a leading cause of nonrelapse mortality (NRM) as well as debilitating morbidity (1–3). Some transplant approaches such as cord blood transplantation (4), T-cell depletion (5), antithymocyte globulin (ATG) or alemtuzumab (6, 7), and posttransplant cyclophosphamide (PTCY) (8) lower, but do not eliminate, the risk of cGVHD. The diagnosis of cGVHD and its severity are usually assessed by nonspecific clinical symptoms in many organs (9) and represent the culmination of tissue perturbations initiated by infusion of donor cells.

Currently, no validated laboratory tests exist to stratify for the likelihood of development of cGVHD in individuals without clinically apparent disease, which is defined as a risk marker according to the Food and Drug biomarkers, endpoints, and other tools (BEST) recommendations (10). Several potential plasma markers of cGVHD have been identified at diagnosis; however, only a panel including STimulation 2 (ST2), chemokine (C-X-C motif) ligand 9 (CXCL9), matrix-metalloproteinase-3 (MMP3), and osteopontin (OPN) has been identified as a potential risk biomarker that could predict future cGVHD occurrence when measured on day 100 after HCT (11). This panel was exclusively tested as risk marker in 87 cGVHD cases that were matched to 93 controls — not in a real world cohort. No discovery using tandem mass spectrometry proteomics for risk biomarkers on multicenter cohort samples has been performed (12). Further, in samples from the Blood and Marrow Transplant Clinical Trials Network (BMTCTN) 0201, NK cell reconstitution depended on graft source (peripheral blood [PB] versus bone marrow [BM]) (13) but no proteomics on graft-specific cGVHD plasma has been performed so far. In the NIH cGVHD consensus series, identifying risk biomarkers was determined to be a priority to enable preemptive treatment (1, 3, 14–17). Further, finding candidate biomarkers in multicenter samples from the BMTCTN will provide definitive evidence of their ability for cGVHD risk stratification.

We studied 8 previously identified diagnostic markers that could represent risk markers and 5 graft-specific markers identified through novel quantitative proteomics and compared day 90 plasma samples from patients who developed cGVHD with those who did not to assess association of markers with occurrence of cGVHD. Plasma samples were from 2 multicenter cohorts for a total of 982 HCT.

## Results

### Identification of graft-specific risk markers via proteomics discovery.

We first compared, using quantitative proteomics, pooled plasma taken on day 90 after HCT from PB recipients who developed cGVHD by day 180, PB recipients who did not develop cGVHD, BM recipients who developed cGVHD by day 180, and BM recipients who did not develop cGVHD. To insulate the early cGVHD proteomic signal from the potential residual acute GVHD (aGVHD) signal, we selected patients that had no aGVHD on day 90 after HCT, never developed aGVHD grade 3–4, were on less than 0.5 mg/kg/day corticosteroids on day 90, or patients who died or relapsed before day 180 after HCT. We then compared, for both PB and BM grafts, patients who developed cGVHD by day 180 after HCT versus patients who never developed cGVHD within a minimum followup of 360 days, (Supplemental Figure 1; supplemental material available online with this article; https://doi.org/10.1172/JCI168575DS1). Proteomics workflow is shown in Supplemental Figure 2. Of 617 proteins identified and quantified, we selected proteins that were increased at least 1.25-fold in patients with cGVHD and had available ELISA for high-throughput quantitative validation. Nine proteins were selected: dermcidin (DCD), chitinase-3-like-1 (CHI3L1), CCL5, fibrinogen-like-2 (FGL2), desmoglein-1 (DSG1), colony stimulating-factor-1 (CSF1), CD276 molecule (B7H3), IL1-receptor-accessory-protein (IL1RAP), and dickkopf-3 (DKK3). Five candidates — CCL5, CSF1, B7H3, IL1RAP, DKK3 — were further evaluated in the validation phase based on their discrimination in individual plasma samples of patients with cGVHD compared with those without cGVHD independent of graft source (Supplemental Figure 3).

### Assessment of 8 previously identified and 5 graft-specific candidate biomarkers of cGVHD risk in PB versus BM recipients in BMTCTN 0201 samples.

The 5 proteomic candidate markers were measured along with 8 previously identified cGVHD markers — ST2, CXCL9, MMP3, OPN, CXCL10, CD163, IL17, and B-cell activating factor (BAFF) (11, 18–23) — in BMTCTN 0201 recipient to assess their association with cGVHD occurrence separately in patients receiving PB and BM. This cohort included all patients with at least a sample available on day 90 after HCT and for some, additional samples available at days 180 and 360 after HCT. As patients were randomized to receive PB or BM, the 2 groups were well balanced for demographic characteristics (Table 1). As previously published for the full randomized cohort, there was no difference in rates of prior aGVHD grade 2–4 in the PB versus BM groups but there was an over-representation of cGVHD in the PB group (Table 2) (24). The cGVHD organ involvement is summarized in Supplemental Table 1 for both cohorts. The organ distribution was close to published cohorts (25, 26).

In univariate analyses, among thirteen markers, CXCL9, DKK3, and IL17 were correlated with cGVHD in patients who received PB graft, while CXCL10 and MMP3 were correlated with cGVHD in patients who received BM graft (Table 3). Of note, marker levels in patients without cGVHD receiving PB as a graft source were not different from marker levels in patients without cGVHD receiving BM as a graft source (Supplemental Table 2). Spearman correlations between CXCL9 and CXCL10 were significant as well as between MMP3, DKK3 and ST2 (Supplemental Table 3), suggesting that multivariate analysis may select only 2 markers but that an alternate model will give similar results. In multivariate analyses, recipients of PB with 1 log_n_ increase in CXCL9 and DKK3 were 1.2 (95%CI: 1.1–1.4, *P* = 0.003) and 2.0 (95%CI: 1.2–3.4, *P* = 0.008) and recipients of BM with 1 log_n_ increase in MMP3 were 1.4 (95%CI: 1.1–1.7, *P* = 0.003) times more likely to develop cGVHD (Table 4). After adjustment for significant clinical covariates (conditioning regimen, and antithymocyte globulin [ATG]), CXCL9 and DKK3 continue to be significantly correlated with risk of developing cGVHD in patients who received PB grafts (Table 4). Additionally, CXCL9, DKK3, and IL-17 were also correlated with extensive cGVHD in univariate analyses in patients who received PB grafts, and CXCL9 and DKK3 remained significant in patients with extensive cGVHD after multivariate analyses and adjustment for clinical covariates (Supplemental Tables 4 and 5). In multivariate analyses for patients who received BM grafts and following adjustment for clinical covariates (conditioning regimen, ATG, and sex mismatch [only for BM]), CXCL10 and MMP3 were independently correlated with cGVHD and with extensive cGVHD in patients who received BM grafts (Table 4 and Supplemental Table 5). Of note, aGVHD grade 2–4 before day 90 for PB and BM did not reach statistical significance.

To better define the potential clinical utility of these markers, we generated ROC curves over time within 2–8 months following the day 90 sample, which was 5–11 months after HCT. The 2-month postsample (or 5 months after HCT) evaluation was chosen based on the first and third quartiles of cGVHD onset (Table 2). We compared multivariable models from biomarkers + clinical covariates to clinical covariates alone. AUCs within 2–8 months after the sample for association with subsequent cGVHD in multivariable models of biomarkers (CXCL9+MMP3+DKK3) showed improvement versus clinical covariates alone (Supplemental Figure 4).

### Biomarkers at day 90 after HCT as risk factors for cGVHD occurrence in an independent contemporary BMTCTN 1202 cohort.

To validate these findings, we next measured the 6 lead candidate markers in 653 patients from BMTCTN 1202, an independent prospective multicenter contemporary cohort. Here, we wanted to measure biomarkers without consideration of some signal noise. Since ATG was a significant clinical covariate in BMTCTN 0201 cohort, as previously observed in other studies at different degree of significance (7, 27, 28), we excluded patients who received T cell depletion with ATG or Alemtuzumab. We also excluded patients who received PTCY and who received cord grafts. To limit the noise from residual or late aGVHD, patients who developed late aGVHD after day 80 after HCT and patients who received more than 1 mg/kg corticosteroids after day 56 after HCT were excluded as well. Patients not excluded with at least 1 sample of plasma available at day 90 after HCT and with cGVHD status available at least in the first year after HCT were included in this cohort (Supplemental Figure 5). There were 525 patients with PB and 128 BM transplants fulfilling the criteria above (Table 1). Of note, the exclusion/inclusion criteria were less stringent than for the proteomics discovery analysis. In this cohort, the patient’s population was older, with 42% of PB recipients being over 60 years of age compared to only 14% in BMTCTN 0201. More reduced intensity conditioning regimen were performed in PB recipients compared with the 0201 cohort (47% versus 24%). Importantly, in this nonrandomized real-life cohort with selection criteria described above, the frequency of prior grade 2–4 aGVHD was similar to the earlier BMTCTN 0201 cohort in PB transplant at approximately 45%, with a slight trend toward less prior aGVHD in the BM group. As expected, there was also a higher incidence of cGVHD within a year in PB transplant (65%) compared with BM transplant (39%) (Table 2). cGVHD severity using the NIH global scoring system was also higher in the PB group; the distribution of organs involved was the same as in the BMTCTN 0201 cohort (Table 2 and Supplemental Table 1).

As in the BMTCTN 0201 cohort, Spearman correlations between CXCL9 and CXCL10 were significant, as well as between MMP3, DKK3, and ST2 (Supplemental Table 6). We performed univariate analyses by graft source, and CXCL9, DKK3, and MMP3 were associated with risk of cGVHD in PB recipients (Table 5). In multivariate analyses, patients receiving PB grafts with 1 log_n_ increase in CXCL9 and MMP3 were 1.1 (95%CI: 1.0–1.2, *P* = 0.015) and 1.2 (95%CI: 1.1–1.4, *P* < 0.001) times more likely to develop cGVHD (Table 6). After adjustments for clinical covariates (GVHD prophylaxis and HLA matching) CXCL9 and MMP3 remained independently significant risk biomarkers (Table 6). Similar results are found with a model including DKK3 instead of MMP3 as they are highly correlated (not shown). In univariate and multivariate analyses, MMP3 was correlated with moderate and severe cGVHD in patients who received PB grafts. Of note, there were no significant clinical covariates to adjust for when considering moderate and severe cGVHD (Supplemental Tables 7 and 8). In patients who received BM grafts, MMP3 was associated with risk of cGVHD in univariate analysis and remained significant in biomarker multivariate analysis and after adjustment for significant clinical covariates (GVHD prophylaxis and HLA matching) (Tables 5 and 6). When models were applied to moderate and severe cGVHD occurrence, CXCL9, MMP3, DKK3, and ST2 were significant in univariate analysis, and CXCL9 and ST2 remained correlated with moderate and severe cGVHD in biomarker multivariate analysis (no significant clinical covariates) (Supplemental Tables 7 and 8).

The biomarkers (CXCL9+MMP3+DKK3) measured at day 90 improve AUCs for risk prediction within 2 months after the sample was taken (5 months after HCT) from 0.64 for clinical covariates to 0.70 in the combined model. In contrast to cohort 0201, this high early predictability decreases at 11 months after HCT (Supplemental Figure 6).

### Performance of biomarkers score in risk prediction for cGVHD occurrence within 2 months and cumulative incidences within 24 months in BMTCTN 0201 and 1202 cohorts.

**Figure 1:**
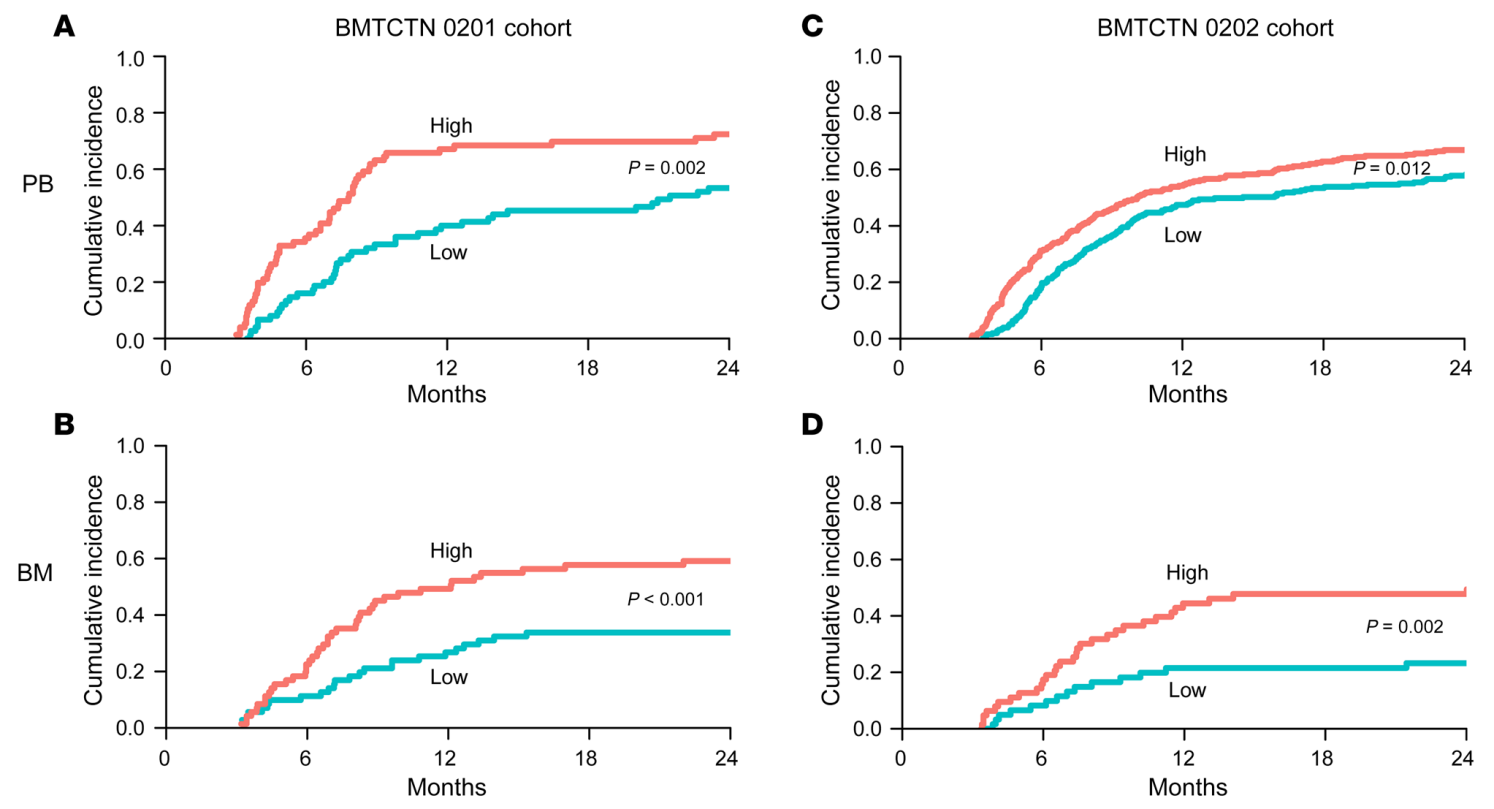
Cumulative incidences of cGVHD by high and low biomarker scores in BMTCTN 0201 and 1202 cohorts for PB and BM recipients. Curves comparing high versus low biomarker scores (above and below the median cutpoint): (**A**) in PB patients from BMTCTN 0201 cohort, score including CXCL9+MMP3+DKK3, *P* = 0.002; (**B**) in BM patients from BMTCTN 0201 cohort, score including MMP3+clinical, *P* < 0.001; (**C**) in PB patients from BMTCTN 1202 cohort, score including CXCL9+MMP3+DKK3, *P* = 0.012; (**D**) in BM patients from BMTCTN 1202 cohort, score including MMP3+clinical, *P* = 0.002.

The incidence of cGVHD at day 150 after HCT (2 months after the sample was taken) was 22% in the BMTCTN 0201 cohort and 15% in BMTCTN 1202 cohort in PB grafts (Table 7). Sensitivity, specificity, positive predictive value (PPV), and negative predictive value (NPV) of the biomarker score (CXCL9+MMP3+DKK3) at different cutpoints for the prediction of risk of cGVHD occurrence within 2 months after the sample was taken in 1202 validation cohort were similar to those of the training cohort. The cutpoints at the median achieved a specificity over 50% with 72% and 75% sensitivities and 88% and 92% NPV in 0201 and 1202 cohorts, respectively (Table 7). Therefore, in BMTCTN 0201, 32% of patients with high biomarkers risk score (greater than the median) were likely to develop cGVHD within 2 months, while only 12% of patients with low biomarkers risk score were likely to develop cGVHD, *P* = 0.002. In BMTCTN 1202, 22% of patients with high biomarker risk scores developed cGVHD within 2 months and only 8% of patients with low biomarker risk scores, *P* < 0.001 developed cGVHD. Cumulative incidences of cGVHD incidence within 24 months in PB recipient by this high and low biomarker score using the median as a cutpoint are shown in Figure 1. For BM recipients, cGVHD cumulative incidence were developed using a score that included MMP3 and clinical covariates (Figure 1).

### Organ-specific associations with biomarker score.

We next examined associations of the biomarkers score with specific organ involvement and found that, overall, the biomarker score measured at day 90 after HCT was not significantly correlated with a particular organ in both cohorts, with an exception of correlation with the gastrointestinal (GI) target in the BMTCTN 1202 cohort (Supplemental Table 9); although the GI distribution was not different between cohorts at approximately 35% and was close to published studies (Supplemental Table 1) (25, 26).

### Validation of CXCL9, DKK3, and MMP3 as risk markers in a murine model of cGVHD.

**Figure 2:**
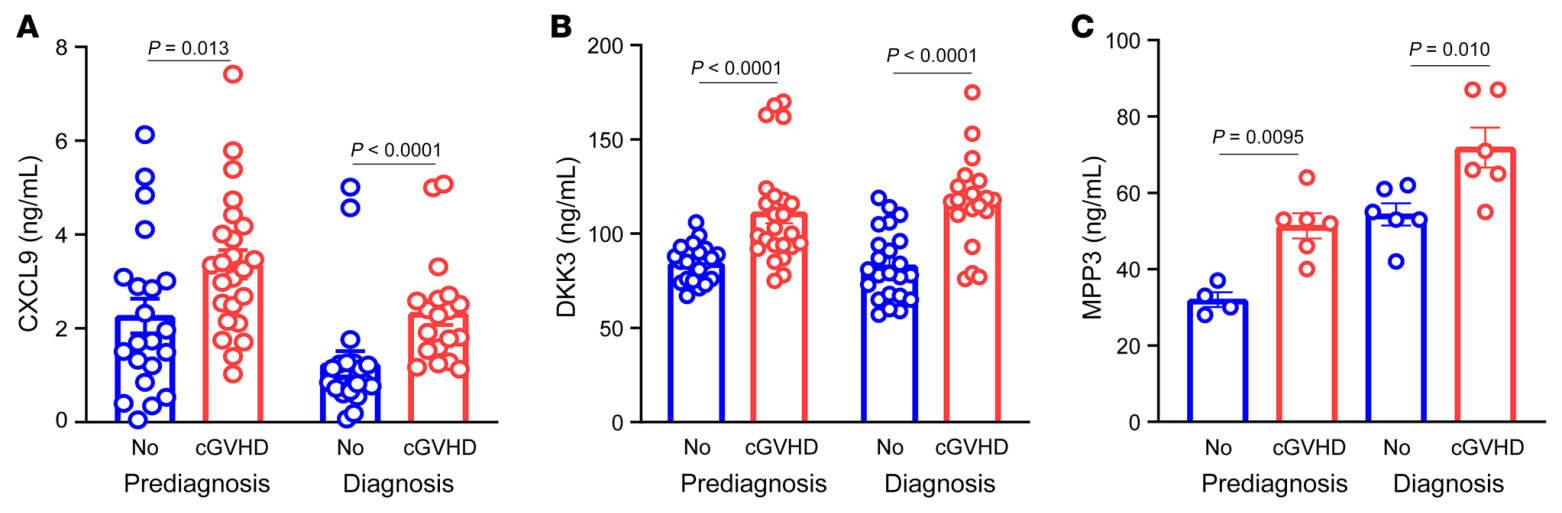
CXCL9, DKK3, and MMP3 circulating concentrations in mice with and without cGVHD before and at diagnosis. (**A**) CXCL9 concentrations in HCT mice with and without cGVHD (before and at diagnosis). B10BR irradiated recipient mice received 10 × 10^6^ B6 T cell depleted BM cells with 7 × 10^5^ T cells (cGVHD) or without (No). Serum was collected at day 18 (prediagnosis) and 28 (diagnosis) after HCT. CXCL9 was measured with ELISA (Raybiotech), data are shown as mean ± SEM, Mann-Whitney test, cGVHD (*n* = 21) and no cGVHD (*n* = 24) in the prediagnosis group, and cGVHD (*n* = 21) and no cGVHD (*n* = 19) in the diagnosis group. (**B**) Using the same model, DKK3 was measured with ELISA (Raybiotech), cGVHD (*n* = 21) and no cGVHD (*n* = 24) in the prediagnosis group, and cGVHD (*n* = 23) and no cGVHD (*n* = 20) in the diagnosis group. (**C**) Using the same model, MMP3 was measured with ELISA (R&D system), cGVHD (*n* = 4) and no cGVHD (*n* = 6) in the prediagnosis group, and cGVHD (*n* = 6) and no cGVHD (*n* = 6) in the diagnosis group.

To examine if these markers would be relevant in reverse translation, we used an established murine model of cGVHD that does not show signs at 19 days and does show signs of cGVHD 28 days after HCT, which we defined as prediagnosis of cGVHD (29–31). This model has been designed so that mice do not develop histopathological or clinical evidence of aGVHD. This is achieved by infusing only approximately 72,000 allogeneic T cells; in the same strain combination, a high level of aGVHD lethality typically requires at least 1,500,000 (> 20-fold higher) and most often 4,500,000–7,500,000 (up to 100-fold higher) T cells with higher dose radiation for greater myeloablation/immune suppression than used in the cGVHD (see initial reports) (32, 33). This low T cell dose in the cGVHD model results in weight loss typically under 10% and at least 85% long-term survival. Consistent with these survival and weight data, histopathology scores show cGVHD without hallmarks of aGVHD when examined at the termination of the experiment. This model has since been considered as a good systemic model of cGVHD without significant overlap with aGVHD. We found in this model that circulating levels of CXCL9, DKK3, and MMP3 were already significantly elevated in day 18 samples, on average 10 days before the diagnosis of cGVHD, including pulmonary fibrosis (Figure 2).

### Day 90 cGVHD biomarkers and NRM.

The biomarkers were primarily evaluated for their value for risk stratification for future occurrence of cGVHD. However, they may also have prognostic value for NRM, which we evaluated. Most causes of deaths from NRM were GVHD-related (Supplemental Table 10). In univariate analyses in BMTCTN 0201, MMP3 and ST2 in PB recipients and MMP3, ST2, and CD163 in BM recipients were associated with NRM (Supplemental Table 11). In multivariate analyses, ST2 in PB and ST2 and CD163 in BM recipients continued to be significant even after clinical covariate adjustments (conditioning regimen in PB and HLA mismatch in BM) (Supplemental Table 12). Patients receiving PB grafts with 1 log_n_ increase in ST2 were 2.1 (95%CI: 1.4–3.2, *P* < 0.001) times more likely to die without relapse. In BMTCTN 1202 and univariate analyses, MMP3, DKK3, and ST2 in PB and CD163, DKK3, and ST2 in BM recipients were associated with NRM (Supplemental Table 13) while only ST2 in PB, and CD163 and DKK3 in BM remained significant after adjustment for clinical covariates (age and donor type in PB and none in BM, Supplemental Table 14). In the BMTCTN 1202 cohort, patients receiving PB grafts with 1 log_n_ increase in ST2 were 1.6 (95%CI: 1.3–2.0, *P* < 0.001) times more likely to die in remission. Cumulative incidences of NRM by high and low ST2 using the upper quartile as a cutpoint are shown in Supplemental Figure 7.

## Discussion

CXCL9, MMP3, and DDK3, when measured on day 90 after HCT — long before any signs of cGVHD — were confirmed as risk biomarkers of cGVHD, in 2 multicenter cohorts totaling approximately 1,000 HCT recipients. This was the largest cohort studied for risk markers of cGVHD and the sole study using all participants’ samples as recommended by NIH cGVHD consensus biomarker working group. Indeed, monitoring for signs of incipient cGVHD is a substantial problem in patients. Current diagnosis is based on clinical signs that may be confirmed by invasive biopsy of skin and appendages, mouth, female genitalia, esophagus, lungs, and connective tissues. Unfortunately, these signs often reveal late-stage fibrotic lesions, as opposed to early lesions that may be more amenable to treatment.

Our discovery approach on samples from day 90 after HCT has highlighted some interesting biologic pathways that may represent novel therapeutic avenues; however, only 1 protein, DKK3, was found to be consistently increased in multivariate analyses in both cohorts. DKK3 was also recently found in a proteomics study using sclerotic cGVHD samples at diagnosis (34), although, our data suggest that DKK3 is a systemic marker. Further, the novelty of our proteomics discovery was to find candidates for risk of developing cGVHD using samples at day 90 after HCT long before the diagnosis of cGVHD is established, while the study mentioned above did proteomics discovery with samples taken at the time of diagnosis of sclerosis on average 12 months (360 days) after HCT and validated in samples taken 29 months (870 days) after HCT. Additionally, our preclinical murine model showed that both DKK3 and MMP3, which are tissue remodeling and fibrotic markers, were elevated prediagnosis before organ damage. As far as we know, it has never been shown that fibrotic markers measured early after HCT have been associated with early risk of cGVHD. Thus, we believe that our findings may contribute to knowledge that was not anticipated based on our current understanding of the biology of cGVHD.

In both cohorts, Spearman correlations between DKK3 and MMP3 were high, resulting in DKK3 and CXCL9 being the best predictors in BMTCTN 0201 and MMP3 and CXCL9 being the best predictors in BMTCTN 1202 in multivariate analyses. Both DKK3 and MMP3 are important as multivariate models because CXCL9 + DKK3 provide similar HR and *P* values as CXCL9 + MMP3. To not lose any information, we decided in our scoring system to include all 3 markers. The biomarker score including CXCL9+MMP3+DKK3 at the median was able to predict future occurrence of cGVHD within 2–8 months from sample acquisition (or 5–11 months after HCT) in both cohorts and with significant differences compared with the score including clinical covariates alone. To our knowledge, this is the first study providing PPV and NPV statistical performances for cGVHD risk biomarkers. This biomarker score may help stratify patients at low and high-risk for cGVHD who may benefit from future preemptive intervention. In the low-risk group, a randomized trial comparing rapid immunosuppression taper to no intervention could be proposed. In the high-risk group, due to the low incidence and PPV, a prospective study with serial biomarkers measurements (every 2–3 months) may increase PPV that will satisfy criteria for a therapeutic intervention.

CXCL9 is a T cell type 1 chemokine detected in the blood that attracts CXCR3^+^ T cells in cGVHD target organs (20, 35). Inhibitors of the CXCL9/CXCR3 axis are under development, such as the small molecule SCH546738 (36). This axis could also be targeted indirectly by molecules such as emapalumab — an anti-IFNγ — and JAK inhibitors that have shown to decrease CXCL9 levels as well as improvement of inflammatory diseases (37, 38).

MMP3, a proteolytic enzyme that degrades components of the extracellular matrix, has been involved in lung fibrosing diseases (39). Early MMP3 detection may suggest subclinical fibrosing disease that might be more apparent in the BM group. MMP3 inhibitors have failed in clinical trials for chronic diseases. However, targeting, for example, the RIPK1 pathway with GSK2982772 in rheumatoid arthritis showed decrease production of MMP3 compared with the placebo group (40) and may be a potential avenue for treatment in cGVHD.

DKK3 — a glycoprotein that regulates Wnt signaling, RYK, and Ror2 (41) — can promote fibrosis (42). Like MMP3, its early detection may suggest subclinical fibrosing disease. A DKK3-blocking monoclonal antibody has been developed and tested in pancreatic ductal adenocarcinoma, a highly fibrogenic cancer (43). Additionally, in the context of cGVHD, Wnt signaling has been shown to be activated in experimental models of sclerotic cGVHD and its specific inhibition ameliorates cGVHD (44).

ELISAs measure soluble ST2 that acts as a decoy receptor for IL-33 (45). ST2 blockade with a neutralizing antibodies or small molecule inhibitors reduced aGVHD severity and mortality (46, 47). ST2 was not a risk marker of cGVHD in this study as opposed to the finding in the Chronic Graft-Versus-Host Disease Consortium (11), possibly due to different cGVHD incidences in the cohorts. Nevertheless, ST2 continues to be a prognostic marker for NRM even after multivariable and clinical adjustments in both cohorts, as shown with early studies (48).

In a reverse translation paradigm, we tested these 3 risk biomarkers in an established cGVHD preclinical model. This bedside-to-bench approach has not been used in previous GVHD biomarkers studies. We selected the alloantibody-driven multiorgan system cGVHD model as a prototype for systemic cGVHD affecting all organs because no specific organ has been involved in the 2 patients cohorts (Supplemental Table 9) (29–31). This model also has a well-characterized kinetic where there are no apparent clinical signs of cGVHD at day 18 after HCT with the onset of clinical signs starting around 28 days. We posited that these markers could also be detected in this murine model and they will be detectable before the clinical signs. Since cGVHD is a relatively acellular process and fibrosis is a dominant feature, elevation of serum proteins of fibrogenesis, such as MMP3 and DKK3, before the clinical signs suggest that fibrosis is anæ early phenomenon during HCT. We found that circulating levels of CXCL9, DKK3, and MMP3 were already significantly elevated in day 18 samples before the diagnosis of cGVHD. Since there are few treatment strategies available that specifically target the pathogenesis of fibrosis, early recognition of fibrogenesis may enable treatments that inhibit upstream immune processes before irreversible tissue damage occurs.

A surprising finding from this analysis is the identification of markers of fibrosis that predate the clinical onset of cGVHD. A possible mechanism is that these biomarkers of early fibrosis are a manifestation of tissue repair from aGVHD or infection, which have been shown as potential risk factor for cGVHD. Previous aGVHD was not a clinical covariate in the present study. Additionally, published proteomics discoveries in patients with aGVHD (samples taken at onset, median day 28 after HCT) do not suggest it is the case as DKK3 was detected but not upregulated in patients with aGVHD and MMP3 was not detected (48–51). Further, in an unpublished proteomics analysis for early sepsis (samples taken at median day 28 after HCT) neither DKK3 nor MMP3 were detected. Furthermore, we looked at infections between days 56 and 90 and correlated infection rates with DKK3 and MMP3 levels and did not find a difference (not shown). A second potential mechanism is that these markers are surrogates of the modulation of mesenchymal stem cells (MSCs). DKK3 has been reported to be secreted by MSCs, which have been shown to limit immune responses, including GVHD, by multiple soluble factors. Since DKK3 also regulates Wnt signaling (42), and Wnt/β-catenin signaling mediates increased osteogenic capacity and decreased adipogenic capacity in MSCs from patients with cGVHD MSCs (52), it is possible that DKK3 marks an early modulation of MSCs function toward a less regulatory phenotype. Thirdly, for alloreactive T cells to infiltrate target tissues, they have to migrate through the extracellular matrix (ECM). MMP-3 not only cleaves ECM structural proteins such as collagen, fibronectin, and laminin, but also activates other MMPs, which are able to cleave and activate of inflammatory mediators such as TNF-α and IL-1β (53). Thus, excess of MMP3 in the plasma may systemically represent the tissue damage created by alloreactive T cells forcing their passage through the ECM. Together, the implication of this study is that, for patients with increase in plasma MPP3 and DKK3, targeting solely the inflammatory response may have limited efficacy. Since MMP3 and DKK3 seem not to be present at the acute phase, early monitoring between the acute phase and the start of the chronic phase (days 56–80 after HCT) could guide an early preemptive intervention.

There are, however, limitations to this study. Only 1 sample at day 90 after HCT was available in the BMTCTN 1202 cohort, and since sampling was focused on aGVHD, collection of cGVHD characteristics, although reviewed by BMTCTN protocol team, were not as granular as in BMTCTN 0201. It was not possible to go back to the centers to verify the full accuracy of the cGVHD grading. The 2 BMTCTN cohorts were conducted in different periods and, although the samples were collected prospectively, retrospectively defined data sets were used to develop prediction models. The metrics we chose to compare models are only a subset of metrics used to evaluate new biomarkers. These cohorts did not include GVHD prophylaxis with PTCY that has shown lower cGVHD incidence than conventional prophylaxis, and it would be interesting to understand the role of these risk biomarkers in patients receiving PTCY.

We conclude that noninvasive assessment of day 90 after HCT CXCL9, DKK3, and MMP3 were better risk factors for cGVHD than significant clinical covariates. The ability to identify high-risk patients using these biomarkers before the development of cGVHD may permit more stringent monitoring and preemptive interventions. ST2 was associated with death without relapse in both cohorts. We believe our results may affect the assessment of cGVHD risk-stratification before its development and prognostic significance; however, a generalizable score for high-risk has yet to be developed.

## Methods

### Eligibility criteria.

Any adult or child who received a HCT in the United States as part as of 2 consecutive BMTCTN 0201 (between March 2004 and September 2009) and BMTCTN 1202 (between June 2013 and December 2018) trials, and for whom a sample at day 90 after transplant was collected. Patients were followed for at least 1 year.

### Samples.

Plasma samples were collected prospectively from patients who underwent HCT 90 days after transplant, before cGVHD development for both cohorts. Additional samples at days 180 and 360 after HCT were collected in BMTCTN 0201. Logistics for sample obtention and processing are detailed in Supplemental Tables 15 and 16.

### BMTCTN 0201 cohort.

A total of 329 randomized patients who received PB versus BM grafts from unrelated-donor transplantation and with at least a sample from 90 days after HCT were included in this cohort. Since the BMTCTN 0201 cohort was a prospective randomized trial with cGVHD as a primary outcome, cGVHD evaluation followed the protocol recommendations (consensus) and the protocol team further centrally adjudicated cGVHD diagnosis, grades, and time to onset for all patients included in this cohort. Maximum cGVHD severity was graded by the limited and extensive score. Patients with relapse were included in the analysis. Patients were divided into 2 groups based on graft source (Table 1).

### Subset of BMTCTN 0201 cohort for discovery proteomics.

To identify both risk biomarkers and risk graft-specific cGVHD biomarkers, and to further obtain biomarker candidates without noise from confounding factors, we selected patients who had no aGVHD on day 90 after HCT, never developed aGVHD grade 3–4, were on less than 0.5 mg/kg/day corticosteroids on day 90, and died or relapsed before day 180 after HCT. We then compared 4 plasma pools taken on day 90 after HCT from PB graft recipients who developed cGVHD by day 180, PB graft recipients who did not develop cGVHD, BM graft recipients who developed cGVHD by day 180, and BM graft recipients who did not develop cGVHD (Supplemental Figures 1 and 2).

### BMTCTN 1202 cohort.

We measured concentrations of cGVHD risk biomarkers that were significant in 653 patients from a contemporary cohort with samples available from day 90 after HCT. Patients were followed under the auspices of the prospective clinical trial with cGVHD diagnosis, grades and onset performed by the sites with a centralized review. Maximum cGVHD severity was graded by the NIH global scoring system (9). Patients with relapse were included in the analysis. Patients were then divided into 2 groups based on graft source (Table 1). Other inclusion/exclusion criteria to remove potential confounding factors were applied as described in the results section.

### Proteomic analysis.

Methods have been previously reported (11, 54) and are summarized in the Supplemental Methods.

### ELISA.

ELISAs were performed blinded from clinical information and reference standard results. ELISA procedures and parameters are described in the Supplemental Methods and Supplemental Table 17.

### Murine preclinical model of cGVHD.

Briefly, the C57BL/6→B10.BR model develops multiorgan system disease, including pulmonary fibrosis, resembling, in some respects, bronchiolar obliterans starting at day 28 after HCT. Both T cells and B cells drive this cGVHD. This model has been previously published (29).

### Statistics.

Cohorts defined by trial protocol and graft type were analyzed separately. Patient characteristics for each cohort were described using frequencies for categorical variables or median (range) for continuous variables. Logarithmic-transformed biomarker values (log_n_) were used in all analyses due to skewed raw values. Associations between biomarkers were described using Spearman rank correlation. Cox regression was used to evaluate the association of biomarkers with cause specific hazard of overall development of cGVHD, overall development of severe cGVHD, or with the hazard of NRM across the time continuum of follow-up. For the BMTCTN 0201 cohort, up to 3 measurements per patient were available and biomarkers were analyzed as time-dependent covariates. Selection of clinical characteristics for multivariable modeling was done using stepwise regression; characteristics for consideration in the modeling include the following: recipient age, race, ethnicity, disease, disease status, HLA matching, conditioning regimen, GVHD prophylaxis, ATG use, donor-recipient sex match, and occurrence of aGVHD grade 2–4 before day 90. Landmark analyses for cGVHD occurrence and NRM included all patients alive and still at risk for each event on day 90 after HCT. Sensitivity analyses of these Cox regression models for cGVHD were conducted where relapse was treated as a competing event for cGVHD, in order to minimize the impact of postrelapse interventions that might affect cGVHD risk. AUCs were estimated using a binary logistic regression model with chronic GVHD within 2 months of the biomarker sample as the event; similar AUCs were estimated at 5 and 8 months after the biomarker measurement. Cumulative incidence for high and low biomarker values was estimated using the Aalen-Johansen method. Association of biomarker score with cGVHD organ involvement among patients experiencing cGVHD was done using the Kruskal-Wallis test. Statistical significance was defined as *P* < 0.05.

### Study approval.

The 2 BMTCTN studies were approved by the IRBs of all BMTCTN participating centers. Informed consent was obtained from all patients or their legal guardians before HCT and sample collection. Animal protocols were approved by IACUC at the University of Minnesota and Medical University of South Carolina.

### Data availability.

The mass spectrometry data and the lists of peptidespectrum-matches and proteins reported by Proteome Discoverer and Mascot are publicly available at MassIVE with the accession number MSV000088585. Patients’ biomarker raw data are available through a data transfer agreement (DTA) with MCW and MUSC; direct inquiries can be directed to SP or BRL. Mouse biomarker raw data are presented in supporting data values. All detection tools are available through commercial vendors.

## Author contributions

BRL and SP wrote the main manuscript text. MF and JK prepared Tables 1–6. AH prepared Supplemental Tables 1 and 2. DF, MRL, and DA prepared Supplemental Tables 3 and 4. FAM and BRB prepared Figure 1. PRG prepared Supplemental Figures 2 and 3. All authors reviewed the manuscript.

## Supplementary Material

Supplemental data

ICMJE disclosure forms

Supporting data values

## Figures and Tables

**Table 7:**
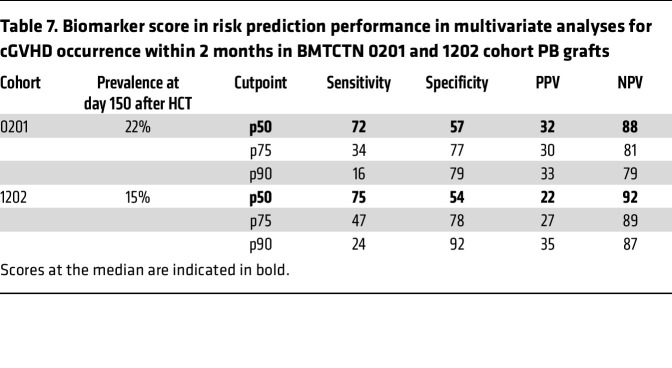
Biomarker score in risk prediction performance in multivariate analyses for cGVHD occurrence within 2 months in BMTCTN 0201 and 1202 cohort PB grafts

**Table 6:**
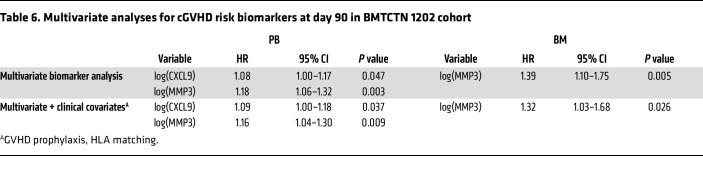
Multivariate analyses for cGVHD risk biomarkers at day 90 in BMTCTN 1202 cohort

**Table 5:**
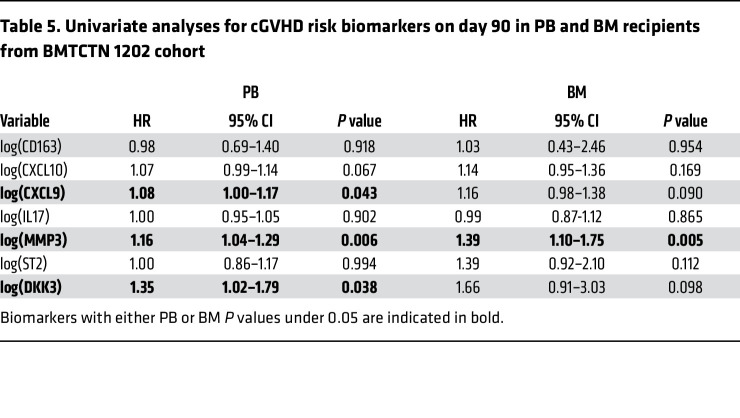
Univariate analyses for cGVHD risk biomarkers on day 90 in PB and BM recipients from BMTCTN 1202 cohort

**Table 4:**
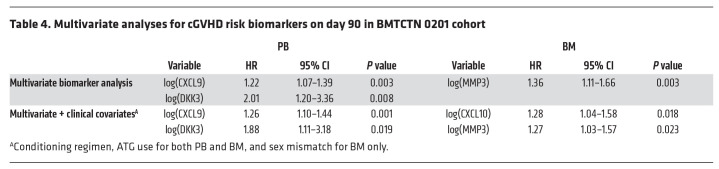
Multivariate analyses for cGVHD risk biomarkers on day 90 in BMTCTN 0201 cohort

**Table 3:**
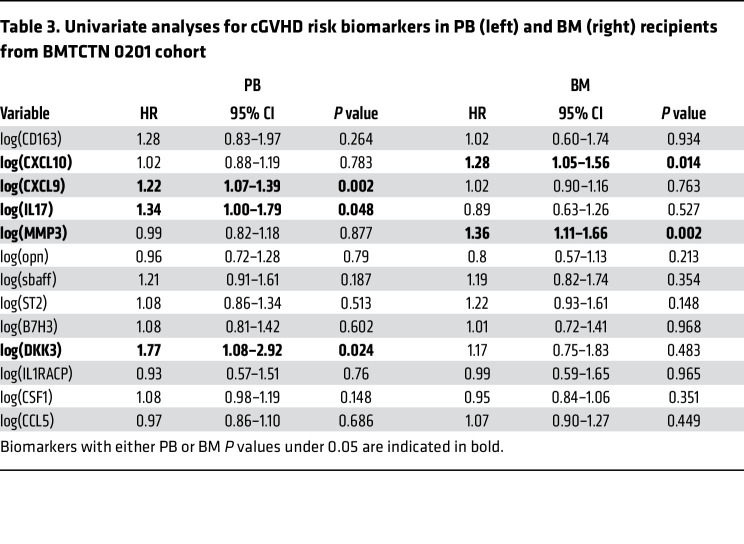
Univariate analyses for cGVHD risk biomarkers in PB (left) and BM (right) recipients from BMTCTN 0201 cohort

**Table 2:**
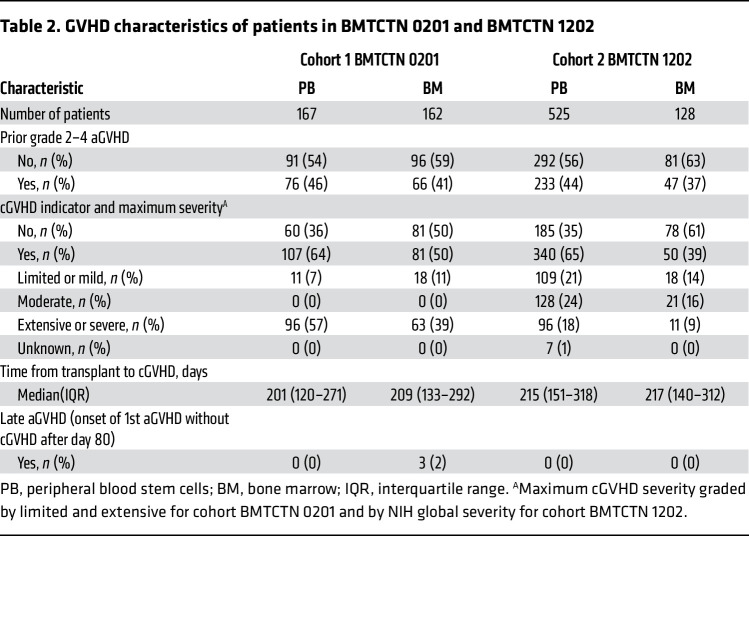
GVHD characteristics of patients in BMTCTN 0201 and BMTCTN 1202

**Table 1:**
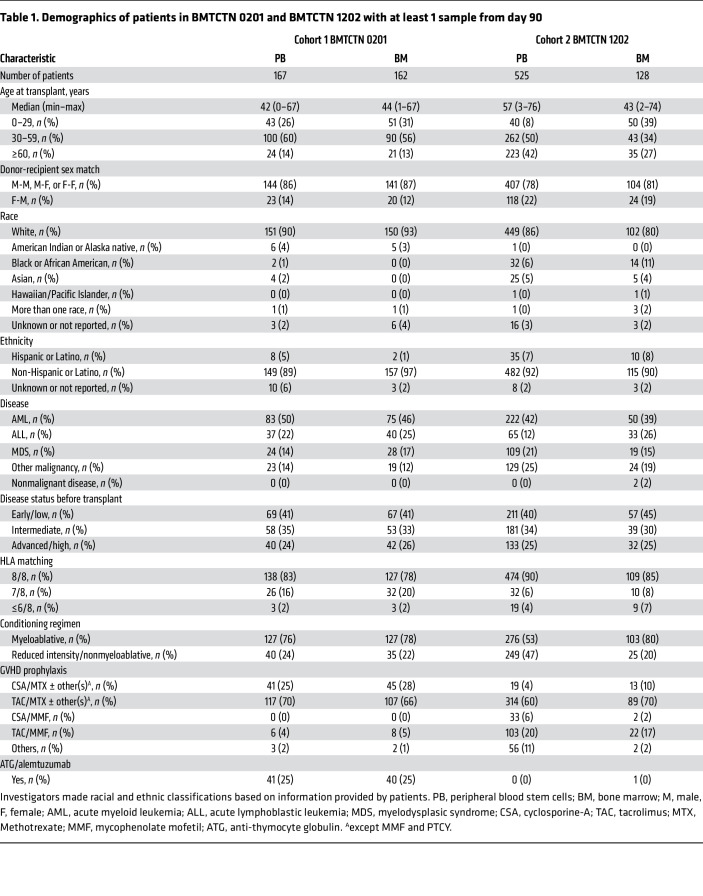
Demographics of patients in BMTCTN 0201 and BMTCTN 1202 with at least 1 sample from day 90

## Version 1 08/01/2023

Electronic publication
