# Supplementary material for: Validated graft-specific biomarkers identify patients at risk for chronic graft-versus-host disease and death
Source: J Clin Invest. 2023 Aug 1;133(15):e168575. doi: 10.1172/JCI168575 (PMC10378149; doi:10.1172/JCI168575)
Supplement: Supplemental data [file jci-133-168575-s065.pdf]

SUPPLEMENTAL MATERIAL

Validated Graft-specific biomarkers identify patients at risk for chronic graft-versus-host disease and death

Brent R. Logan, Denggang Fu, Alan Howard, Mingwei Fei, Jianqun Kou, Morgan R. Little, Djamilatou Adom, Fathima A. Mohamed, Bruce R. Blazar, Phil R. Gafken, and Sophie Paczesny

Table of Contents

Supplemental methods

Figure S1. BMTCTN 0201 Patients' selection for proteomics
Figure S2. Proteomics workflow
Figure S3. Candidate Proteins not previously identified in a proteomics workflow with ELISA tested for their validity and accuracy
Figure S4. AUCs within 2 months post day 90-sample for association with subsequent cGVHD in multivariable models of clinical covariates + Biomarkers (CXCL9 + MMP3 + DKK3) versus clinical covariates alone in BMTCTN 0201 cohort
Figure S5. BMTCTN 1202 Patients' selection for cGVHD risk markers validation
Figure S6. AUCs within 2 months post day 90-sample for association with subsequent cGVHD in multivariable models of clinical covariates + Biomarkers (CXCL9 + MMP3 + DKK3) versus clinical covariates alone in BMTCTN 1202 cohort
Figure S7. Cumulative incidences of NRM by high and low ST2 in BMTCTN 0201 and 1202 cohorts

Table S1. Distribution of cGVHD organ involvement
Table S2. Biomarker levels at day 90 for no cGVHD BM vs. no cGVHD PB patients in BMTCTN 0201 cohort
Table S3. Spearman correlation of marker concentrations in BMTCTN 0201 cohort
Table S4. Univariate biomarker analyses for risk biomarkers of extensive cGVHD in PB (left) and BM (right) recipients from BMTCTN 0201 cohort
Table S5. Multivariate analyses for risk biomarkers of extensive cGVHD in BMTCTN 0201 cohort
Table S6. Spearman Correlation of marker concentrations in BMTCTN 1202 cohort
Table S7. Univariate analyses for risk biomarkers of moderate + severe cGVHD in PB (left) and BM (right) recipients from BMTCTN 1202 cohort
Table S8. Multivariate analyses for risk biomarkers of moderate + severe cGVHD in BMTCTN 1202 cohort
Table S9. Organ specific associations with biomarker score
Table S10. Causes of death for Non-Relapse Mortality

Table S11. Univariate analyses for correlation of cGVHD biomarkers with NRM in BMTCTN 0201 cohort
Table S12. Multivariate analyses for correlation of cGVHD biomarkers and NRM in BMTCTN 0201 cohort
Table S13. Univariate analyses for correlation of cGVHD biomarkers and NRM in BMTCTN 1202 cohort
Table S14. Multivariate analyses for correlation of cGVHD biomarkers and NRM in BMTCTN 1202 cohort
Table S15. Details of samples' collection and processing BMTCTN 0201 cohort
Table S16. Details of samples' collection and processing BMTCTN 1202 cohort
Table S17. Details of ELISAs used for candidate protein testing

Supplemental Material references

Supplementary Methods

Proteomic analysis

Four samples containing pooled plasma/sera were sent to Fred Hutchinson Cancer Research Center Proteomics core (patients' selection in Figure S1). Each sample was depleted of the six most abundant serum proteins using a multiple affinity removal (MARs 6) column (Agilent). Protein samples were adjusted to 8 M urea and 50 mM ammonium bicarbonate, reduced with dithiotheritol, alkylated with chloroacetamide, and digested first with Lys-C at a 1:15 enzyme to substrate ratio for 4 hours at 37°C, followed by digestion overnight with trypsin at a 1:20 enzyme to substrate ratio. The resulting peptide samples were desalted using Oasis C18 cartridges (Waters), dried by vacuum centrifugation, and labeled with TMT reagent (ThermoFisher Scientific) with the following labeling assignments (Figure S2):

After labeling, the samples were mixed, desalted, and taken to dryness by vacuum centrifugation. The mixture was fractionated by basic reverse phase chromatography using a 2.1 mm x 15 cm C18 Zorbax Extend column (Agilent) and collecting 80 fractions that were combined into 33 pools using a concatenated pattern. The 33 pools were dried by vacuum centrifugation and resuspended in 40 µL of LC MS/MS loading buffer. Approximately 4 µL of each pool was injected into an Easy nanoLC 1000 (ThermoFisher Scientific) HPLC connected to an OrbiTrap Fusion (ThermoFisher Scientific) mass spectrometer. In-line chromatographic separations into the mass spectrometer were carried out using a 75 µm i.d. x 40 cm column (New Objective, Littleton, MA), packed with Magic C18 AQ 5 µm diameter, 100 Å pore size (Bruker, Billerica, MA), packing material and maintained at 40 °C. The column was directly mounted to the electrospray ion source. Peptide samples were loaded directly onto the column and eluted with a gradient of acetonitrile in 0.1% aqueous formic acid from 2% to 5% over 5 min, then to 30% over 180 min, then to 50% over 10 min and held there for 2 min, followed by a 2 min gradient to 90% and held there for 10 min, at a flow rate of 300 nL/min. The mass spectrometer was operated using a 3 second cycle time. MS1 OrbiTrap resolution was set to 120K (m/z 200) with a maximum injection time of 50 ms and an AGC target of 4E5. MS2 OrbiTrap resolution was set to 15K with a maximum injection time of 160 ms and an AGC target of 5E4. Dynamic exclusion was enabled with a duration of 30 s and precursor charge states from 2-7 were selected for MS/MS. Quadrupole isolation was set

to 1.2 FWHM and higher energy collision dissociation (HCD) was used for fragmentation at a collision energy of 40%.

Mass spectrometry data were analyzed with Proteome Discoverer v2.2.0.388 using a human UniProt protein database (UP000005640, May 23, 2017) along with common contaminants (cRAPome Jan 29, 2015). Searches were performed with settings for the proteolytic enzyme trypsin and maximum missed cleavages was set to 2. The precursor ion tolerance was set to 10 ppm and the fragment ion tolerance was set to 0.6 Da. Dynamic modifications included oxidation on methionine (+15.995 Da) carbamidomethyl on cysteine (+57.021 Da), TMT6plex on lysine (+229.163 Da) and N-terminal peptide modification with TMT6plex (+229.163 Da) and N-terminal protein modification of acetyl (+42.011 Da). All search results were run through Percolator for peptide validation and the peptide results were filtered to a 1% false discovery rate. A total of 615 proteins were both identified and had quantification data recorded for each labeling reagent (no proteins with missing data).

Data availability: The mass spectrometry data and the lists of peptide-spectrum-matches and proteins reported by Proteome Discoverer and Mascot are publicly available at MassIVE with the accession number MSV000088585.

High-throughput ELISA

Antibody pairs were purchased as described in Table S1. Proteins were measured in samples using commercially available enzyme-linked immunosorbent assays (ELISA) and following the manufacturer' recommendations and using a sequential ELISA approach previous described.[1-3] All samples and standards were tested in duplicate. All washes were performed using the Aquamax 2000 plate washer (Molecular Devices, Sunnyvale, CA). Absorbance was measured immediately after termination of the substrate reaction using a SpectraMax M2*e* plate reader and results were calculated using SoftMax Pro Version 5.4 (Molecular Devices, Sunnyvale, CA).

Figure S1. BMTCTN 0201 Patients' selection for proteomics. Diagram illustrating the patients' selection for discovery cohort were day 90 post-HCT samples were selected using extreme phenotypes and without previous acute GVHD or acute GVHD grade I-II before day 60.

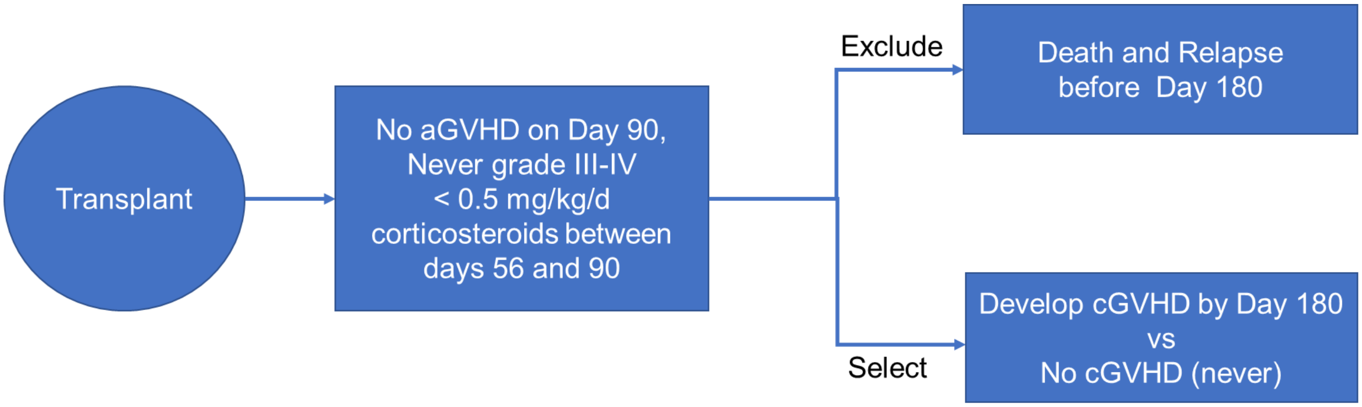

Figure S2. Proteomics workflow.

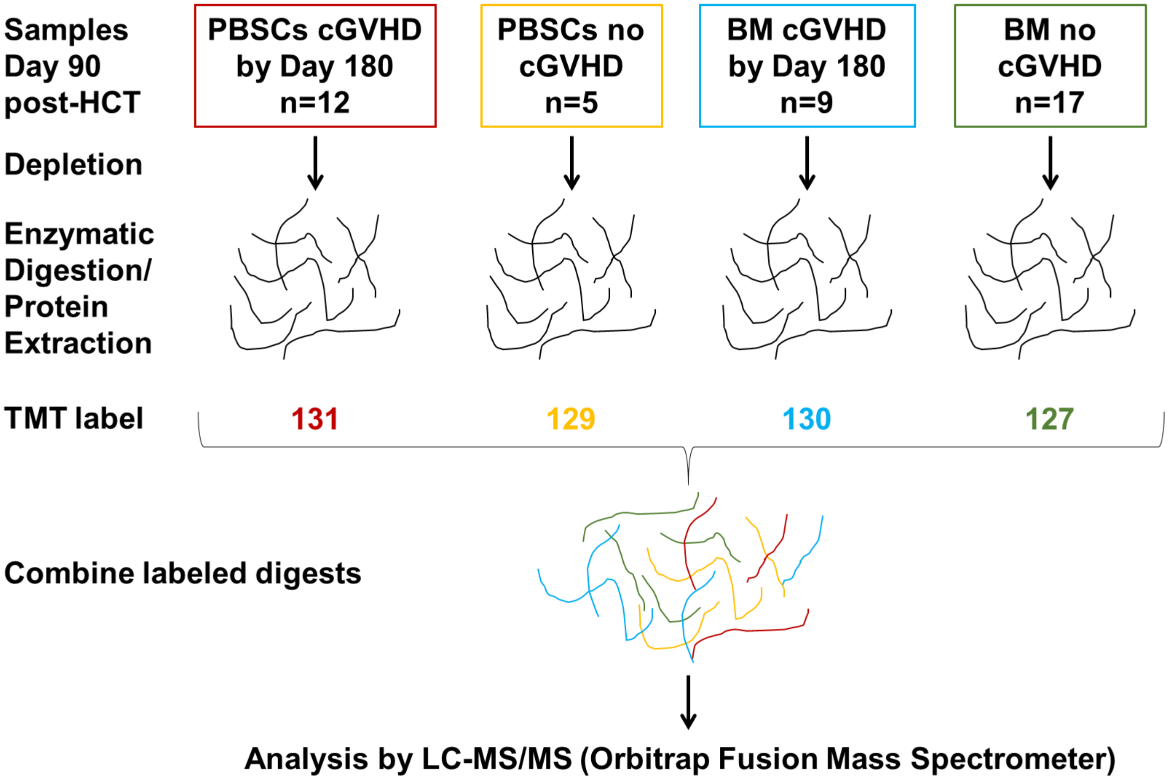

Figure S3. Candidate Proteins not previously identified in a proteomics workflow with ELISA tested for their validity and accuracy.

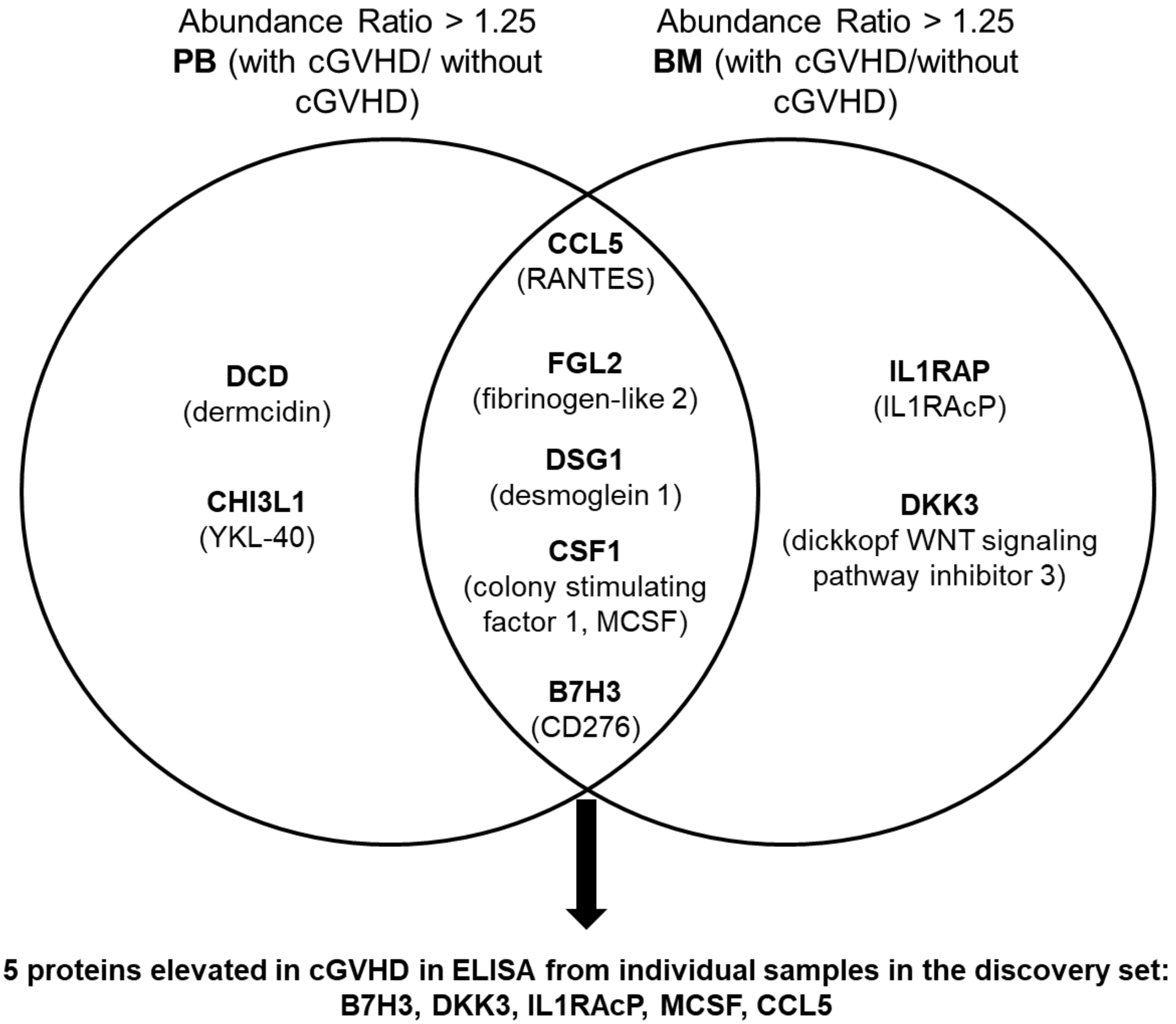

Figure S4. AUCs within 2 to 8 months post day 90-sample for association with subsequent cGVHD in multivariable models of clinical covariates + Biomarkers (CXCL9 + MMP3 + DKK3) versus clinical covariates alone in BMTCTN 0201 cohort

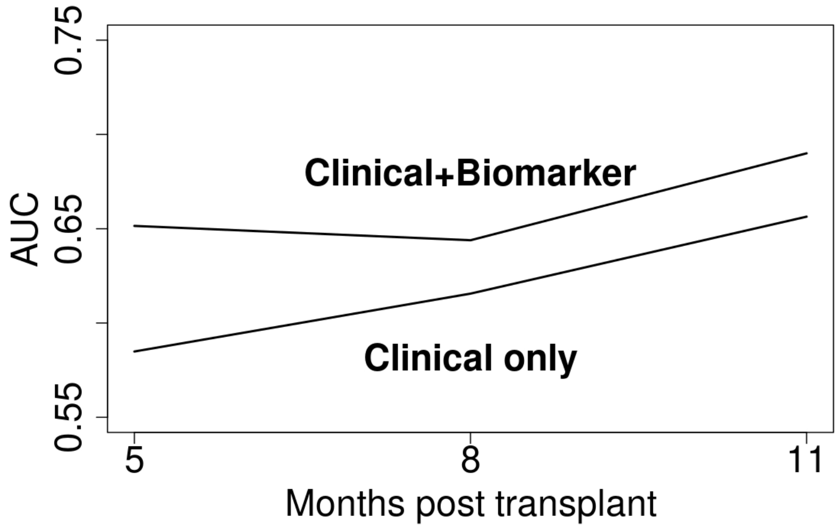

Figure S5. BMTCTN 1202 Patients' selection for cGVHD risk markers validation

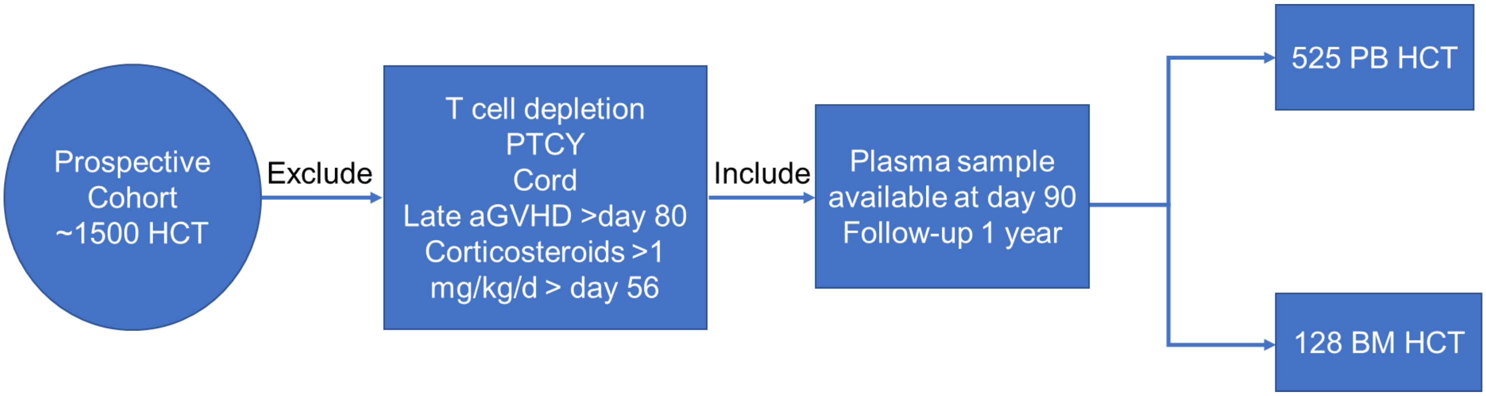

Figure S6. AUCs within 2 to 8 months post day 90-sample for association with subsequent cGVHD in multivariable models of clinical covariates + Biomarkers (CXCL9 + MMP3 + DKK3) versus clinical covariates alone in BMTCTN 1202 cohort

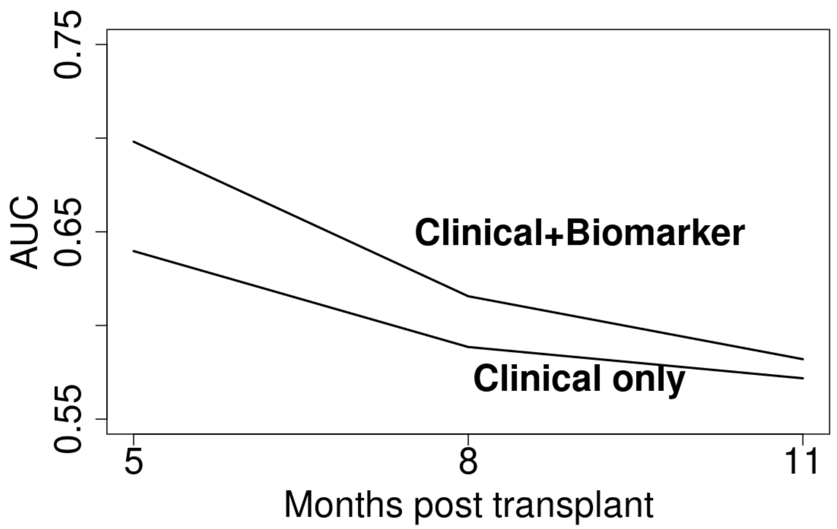

Figure S7. Cumulative incidences of NRM by high and low ST2 in BMTCTN 0201 and 1202 cohorts. Curves comparing high vs low ST2 (above and below the 75th percentile cutpoint): (A) in PB patients from BMTCTN 0201 cohort, p=0.008; (B) in BM patients from BMTCTN 0201 cohort, p=0.011; (C) in PB patients from BMTCTN 1202 cohort, p=0.066; (D) in BM patients from BMTCTN 1202 cohort, p=0.064

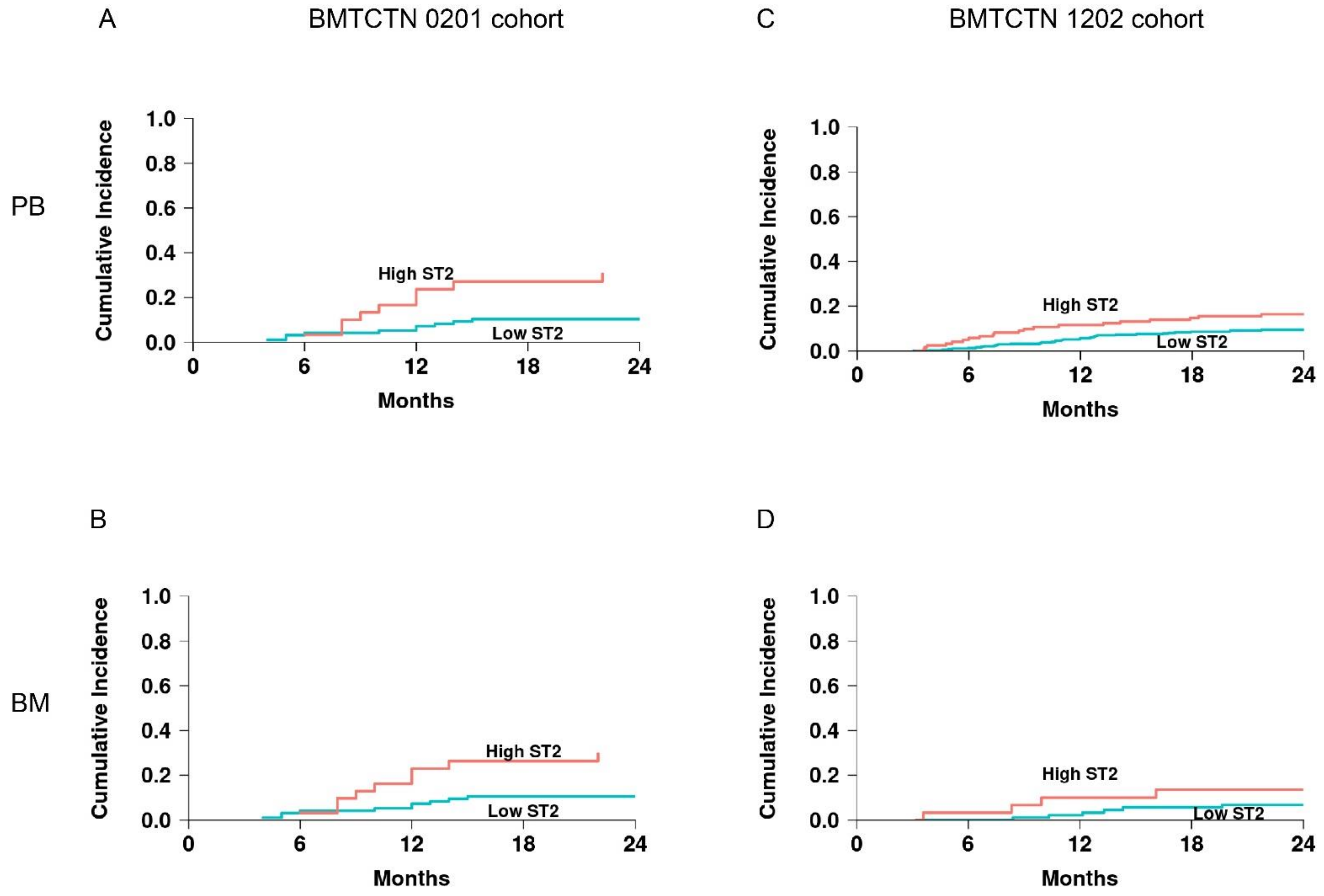

Table S1. Distribution of cGVHD organ involvement

	Cohort 1 BMTCTN 0201		Cohort 2 BMTCTN 1202	
**Characteristic**	**Peripheral Blood Stem Cells (PB)**	**Bone Marrow (BM)**	**Peripheral Blood Stem Cells (PB)**	**Bone Marrow (BM)**
**cGVHD indicator** - no. (%)				
No	60 (36)	81 (50)	185 (35)	78 (61)
Yes	107 (64)	81 (50)	340 (65)	50 (39)
**cGVHD organ involvement**- no. (%)				
Skin	77 (72)	60 (74)	245 (72)	36 (72)
Eyes	59 (55)	31 (38)	209 (61)	21 (42)
Mouth	73 (68)	48 (59)	230 (68)	33 (66)
Lung	26 (24)	23 (28)	92 (27)	12 (24)
GI	38 (36)	30 (37)	111 (33)	15 (30)
Liver	40 (37)	30 (37)	163 (48)	23 (46)
GU	6 (6)	6 (7)	36 (11)	4 (8)
Musculo	10 (9)	4 (5)	51 (15)	6 (12)
Hematological	27 (25)	16 (20)	75 (22)	17 (34)
Other	31 (29)	15 (19)	76 (22)	9 (18)

Table S2. Biomarker levels at day 90 for no cGVHD BM vs. no cGVHD PB patients from BMTCTN 0201 cohort

Variable	PB	BM	P Value
No. of patients	60	81	
CD163 - median (min-max)	609 (196-1372)	673 (226-1688)	0.12[a]
CXCL10 - median (min-max)	0 (0-1)	0 (0-2)	0.88[a]
CXCL9 - median (min-max)	1 (0-20)	1 (0-27)	0.14[a]
IL17 - median (min-max)	0 (0-7)	0 (0-8)	0.52[a]
MMP3 - median (min-max)	19 (2-148)	14 (1-134)	0.12[a]
opn - median (min-max)	135 (19-344)	102 (17-725)	0.07[a]
sbaff - median (min-max)	3628 (81-12829)	3980 (520-26491)	0.18[a]
ST2 - median (min-max)	32 (7-171)	32 (4-186)	0.95[a]
B7H3 - median (min-max)	62 (20-183)	65 (23-1289)	0.52[a]
DKK3 - median (min-max)	70 (21-215)	77 (34-1008)	0.17[a]
IL1RAcP - median (min-max)	167 (81-413)	158 (77-596)	0.83[a]
CCL5 - median (min-max)	20 (0-164)	8 (0-172)	0.05[a]

[a] Kruskal-Wallis test

Table S3. Spearman Correlation of marker Concentrations at day 90 post-HCT in BMTCTN 0201 cohort

Spearman Correlation Coefficients
Prob > |r| under H0: Rho=0

	**IL17**	**CXCL9**	**CXCL10**	**ST2**	**OPN**	**MMP3**	**sBAFF**	**CD163**	**CSF1**	**DKK3**	**CCL5**	**IL1RAcP**	**B7H3**
**IL17**	1.00	0.25<br><.0001	0.27<br><.0001	-0.18<br>0.0021	0.06<br>0.2965	-0.16<br>0.0054	0.29<br><.0001	0.15<br>0.0100	0.12<br>0.0404	0.16<br>0.0046	0.03<br>0.5511	-0.06<br>0.2796	0.19<br>0.0010
**CXCL9**	0.25<br><.0001	1.00	**0.39**<br>**<.0001**	-0.07<br>0.2586	0.08<br>0.1663	-0.10<br>0.0851	0.39<br><.0001	0.32<br><.0001	-0.03<br>0.6201	0.006<br>0.9124	-0.07<br>0.1968	-0.15<br>0.0106	0.10<br>0.0912
**CXCL10**	0.27<br><.0001	**0.39**<br>**<.0001**	1.00	-0.03<br>0.6273	0.005<br>0.9285	-0.002<br>0.9662	0.27<br><.0001	0.24<br><.0001	0.02<br>0.7478	0.12<br>0.0413	0.02<br>0.6710	-0.03<br>0.6312	0.05<br>0.4130
**ST2**	-0.18<br>0.0021	-0.07<br>0.258	-0.03<br>0.6273	1.00	0.18<br>0.0015	**0.63**<br>**<.0001**	-0.40<br><.0001	0.21<br>0.0002	-0.03<br>0.5567	**0.30**<br>**<.0001**	-0.35<br><.0001	0.04<br>0.4663	0.02<br>0.6747
**OPN**	0.06<br>0.2965	0.08<br>0.1663	0.005<br>0.9285	0.18<br>0.0015	1.00	0.11<br>0.0564	0.07<br>0.2012	0.13<br>0.0192	0.008<br>0.8917	0.27<br><.0001	-0.03<br>0.5897	0.03<br>0.5942	0.16<br>0.0054
**MMP3**	-0.16<br>0.0054	-0.10<br>0.0851	-0.002<br>0.9662	**0.63**<br>**<.0001**	0.11<br>0.0564	1.00	-0.46<br><.0001	-0.02<br>0.7592	0.09<br>0.1026	**0.36**<br>**<.0001**	-0.23<br><.0001	0.22<br><.0001	0.09<br>0.1278
**sBAFF**	0.29<br><.0001	0.39<br><.0001	0.27<br><.0001	-0.40<br><.0001	0.07<br>0.2012	-0.46<br><.0001	1.00	0.21<br>0.0002	0.06<br>0.3245	-0.08<br>0.1861	0.03<br>0.5469	-0.13<br>0.0236	0.22<br>0.0002
**CD163**	0.15<br>0.0100	**0.32**<br>**<.0001**	0.24<br><.0001	0.21<br>0.0002	0.13<br>0.0192	-0.02<br>0.7592	0.21<br>0.0002	1.00	0.17<br>0.0037	0.26<br><.0001	-0.16<br>0.0062	-0.11<br>0.0568	0.31<br><.0001
**CSF1**	0.12<br>0.0404	-0.03<br>0.6201	0.02<br>0.7478	-0.03<br>0.5567	0.008<br>0.8917	0.09<br>0.1026	0.06<br>0.3245	0.17<br>0.0037	1.00	-0.18<br>0.0018	0.01<br>0.8220	0.17<br>0.0027	0.71<br><.0001
**DKK3**	0.16<br>0.0046	0.006<br>0.9124	0.12<br>0.0413	**0.30**<br>**<.0001**	0.27<br><.0001	**0.36**<br>**<.0001**	-0.08<br>0.1861	0.26<br><.0001	-0.18<br>0.0018	1.00	-0.18<br>0.0018	0.25<br><.0001	0.36<br><.0001
**CCL5**	0.03<br>0.5511	-0.07<br>0.1968	0.02<br>0.6710	-0.35<br><.0001	-0.03<br>0.5897	-0.23<br><.0001	0.03<br>0.5469	-0.16<br>0.0062	0.01<br>0.8220	-0.18<br>0.0018	1.00	0.001<br>0.9857	-0.07<br>0.2008
**IL1RAcP**	-0.06<br>0.2796	-0.15<br>0.0106	-0.03<br>0.6312	0.04<br>0.4663	0.03<br>0.5942	0.22<br><.0001	-0.13<br>0.0236	-0.11<br>0.0568	0.17<br>0.0027	0.25<br><.0001	0.001<br>0.9857	1.00	0.05<br>0.3609
**B7H3**	0.19<br>0.0010	0.10<br>0.0912	0.05<br>0.4130	0.02<br>0.6747	0.16<br>0.0054	0.09<br>0.1278	0.22<br>0.0002	0.31<br><.0001	0.71<br><.0001	0.36<br><.0001	-0.07<br>0.2008	0.05<br>0.3609	1.00

Table S4. Univariate biomarker analyses for risk biomarkers of extensive cGVHD in PB (left) and BM (right) recipients from BMTCTN 0201 cohort

	PB			BM		
**Variable**	**HR**	**95% CI**	**p-value**	**HR**	**95% CI**	**p-value**
log(CD163)	1.39	0.86-2.25	0.180	1.14	0.62-2.10	0.683
**log(CXCL10)**	1.10	0.93-1.29	0.266	**1.47**	**1.14-1.88**	**0.003**
**log(CXCL9)**	**1.35**	**1.16-1.57**	**<0.001**	1.08	0.93-1.25	0.325
**log(IL17)**	**1.35**	**1.01-1.81**	**0.044**	0.93	0.63-1.36	0.701
**log(MMP3)**	1.00	0.82-1.21	0.986	**1.45**	**1.15-1.82**	**0.001**
log(opn)	1.02	0.75-1.39	0.888	0.76	0.51-1.12	0.162
log(sbaff)	1.26	0.92-1.74	0.151	1.17	0.76-1.81	0.462
log(ST2)	1.10	0.87-1.39	0.440	1.25	0.92-1.70	0.158
log(B7H3)	1.04	0.76-1.42	0.829	1.11	0.77-1.60	0.562
**log(DKK3)**	**1.99**	**1.17-3.40**	**0.011**	**1.66**	**1.05-2.64**	**0.03**
log(IL1RACP)	1.02	0.60-1.72	0.939	1.27	0.73-2.19	0.395
log(CSF1)	1.05	0.94-1.17	0.425	1.01	0.89-1.16	0.849
log(CCL5)	0.88	0.77-1.01	0.076	1.09	0.89-1.32	0.396

Table S5. Multivariate analyses for risk biomarkers of extensive cGVHD in BMTCTN 0201 cohort

PB				BM			
**Multivariate Biomarker Analysis**							
**Variable**	**HR**	**95% CI**	**p-value**	**Variable**	**HR**	**95% CI**	**p-value**
log(CXCL9)	**1.29**	**1.12-1.49**	**<0.001**	log(MMP3)	**1.49**	**1.18-1.87**	**<0.001**
log(DKK3)	**2.18**	**1.27-3.74**	**0.005**	log(CXCL10)	**1.50**	**1.15-1.95**	**0.003**
**Multivariate + clinical covariates** (conditioning regimen, and ATG use for both PB and BM, and sex mismatch for BM only)							
**Variable**	**HR**	**95% CI**	**p-value**	**Variable**	**HR**	**95% CI**	**p-value**
log(CXCL9)	**1.33**	**1.15-1.55**	**<0.001**	log(MMP3)	**1.38**	**1.08-1.76**	**0.009**
log(DKK3)	**2.06**	**1.19-3.57**	**0.01**	log(CXCL10)	**1.47**	**1.13-1.93**	**0.005**

Table S6. Spearman Correlation of marker Concentrations at day 90 post-HCT in BMTCTN 1202 cohort

Spearman Correlation Coefficients<br>Prob > |r| under H0: Rho=0	CXCL10	CXCL9	IL17	MMP3	ST2	DKK3	CD163
**CXCL10**	1.00	**0.40**<br>**<.0001**	0.20<br><.0001	-0.003<br>0.94	0.03<br>0.41	0.02<br>0.61	0.12<br>0.003
**CXCL9**	**0.40**<br>**<.0001**	1.00	0.17<br><.0001	-0.10<br>0.01	-0.05<br>0.20	0.04<br>0.36	**0.30**<br>**<.0001**
**IL17**	0.20<br><.0001	0.17<br><.0001	1.00	-0.05<br>0.23	-0.04<br>0.28	0.06<br>0.11	0.03<br>0.47
**MMP3**	-0.003<br>0.94	-0.10<br>0.01	-0.05<br>0.23	1.00	**0.52**<br>**<.0001**	**0.39**<br>**<.0001**	-0.11<br>0.01
**ST2**	0.03<br>0.41	-0.05<br>0.20	-0.04<br>0.28	**0.52**<br>**<.0001**	1.00	**0.37**<br>**<.0001**	0.15<br><0.001
**DKK3**	0.02<br>0.61	0.04<br>0.36	0.06<br>0.11	**0.39**<br>**<.0001**	**0.37**<br>**<.0001**	1.00	0.12<br>0.002
**CD163**	0.12<br>0.003	**0.30**<br>**<.0001**	0.03<br>0.47	-0.11<br>0.0067	0.15<br><0.001	0.12<br>0.002	1.00

Table S7. Univariate analyses for risk biomarkers of moderate + severe cGVHD in PB (left) and BM (right) recipients from BMTCTN 1202 cohort

	PB			BM		
**Variable**	**HR**	**95% CI**	**p-value**	**HR**	**95% CI**	**p-value**
log(CD163)	0.90	0.58-1.38	0.622	2.66	0.77-9.13	0.120
log(CXCL10)	1.05	0.96-1.14	0.284	1.23	0.96-1.58	0.097
**log(CXCL9)**	1.03	0.94-1.12	0.487	**1.28**	**1.01-1.63**	**0.040**
log(IL17)	0.98	0.93-1.04	0.522	0.93	0.79-1.08	0.338
**log(MMP3)**	**1.19**	**1.05-1.34**	**0.006**	**1.52**	**1.14-2.03**	**0.005**
**log(ST2)**	1.08	0.90-1.29	0.419	**2.04**	**1.27-3.28**	**0.003**
**log(DKK3)**	1.31	0.94-1.82	0.109	**2.62**	**1.34-5.11**	**0.005**

Table S8. Multivariate analyses for risk biomarkers of moderate + severe cGVHD in BMTCTN 1202 cohort

PB				BM			
**Multivariate Biomarker Analysis**							
**Variable**	**HR**	**95% CI**	**p-value**	**Variable**	**HR**	**95% CI**	**p-value**
**log(MMP3)**	**1.18**	**1.05-1.34**	**0.007**	**log(CXCL9)**	**1.28**	**1.01-1.61**	**0.038**
				**log(ST2)**	**2.00**	**1.24-3.21**	**0.004**
**Multivariate + clinical covariates** (N/A: No significant clinical covariates)							
**Variable**	**HR**	**95% CI**	**p-value**	**Variable**	**HR**	**95% CI**	**p-value**

Table S9. Organ specific associations with biomarker score

	BMTCTN 0201 cohort			BMTCTN 1202 cohort		
**Organ**	**Involvement**	**N**	**p-value**	**Involvement**	**N**	**p-value**
Skin	No	25	0.208	No	91	0.681
	Yes	70		Yes	236	
Eyes	No	42	0.822	No	127	0.753
	Yes	53		Yes	200	
Mouth	No	30	0.118	No	109	0.893
	Yes	65		Yes	218	
Lung	No	70	0.571	No	240	0.085
	Yes	25		Yes	87	
GI	No	63	0.399	No	220	0.036
	Yes	32		Yes	107	
Liver	No	60	0.89	No	171	0.338
	Yes	35		Yes	156	
GU	No	90	0.286	No	297	0.154
	Yes	5		Yes	30	
Musculo	No	85	0.389	No	277	0.16
	Yes	10		Yes	50	
Hematologic	No	73	0.784	No	256	0.494
	Yes	22		Yes	71	

Table S10. Causes of death for Non-Relapse Mortality

	Cohort 1 BMTCTN 0201		Cohort 2 BMTCTN 1202	
**Characteristic**	**Peripheral Blood Stem Cells (PB)**	**Bone Marrow (BM)**	**Peripheral Blood Stem Cells (PB)**	**Bone Marrow (BM)**
**Non-relapse mortality** - no. (%)				
No	128 (77)	138 (85)	440 (84)	115 (90)
Yes	39 (23)	24 (15)	85 (16)	13 (10)
**Causes of death** - no. (%)				
GVHD	33 (85)	16 (67)	28 (33)	3 (23)
GVHD + Infection	0 (0)	0 (0)	6 (7)	2 (15)
GVHD + ARDS	0 (0)	0 (0)	2 (2)	0 (0)
Organ failure	1 (3)	1 (4)	9 (11)	0 (0)
ARDS	0 (0)	0 (0)	4 (5)	2 (15)
Infection	3 (8)	2 (8)	15 (18)	1 (8)
Infection + ARDS	0 (0)	0 (0)	3 (4)	0 (0)
Graft failure	0 (0)	2 (8)	0 (0)	0 (0)
Secondary malignancy	0 (0)	1 (4)	3 (4)	2 (15)
Vascular	0 (0)	1 (4)	0 (0)	0 (0)
Autologous recovery	0 (0)	1 (4)	0 (0)	0 (0)
Bleeding	0 (0)	0 (0)	3 (4)	0 (0)
Accident/suicide	0 (0)	0 (0)	3 (4)	0 (0)
Other/Unknown	2 (5)	0 (0)	9 (11)	3 (23)

Table S11. Univariate analyses for correlation of cGVHD biomarkers with NRM in BMTCTN 0201 cohort

	PB			BM		
**Variable**	**HR**	**95% CI**	**p-value**	**HR**	**95% CI**	**p-value**
**log(CD163)**	2.04	0.95-4.40	0.068	**4.66**	**1.57-13.82**	**0.006**
log(CXCL10)	1.09	0.85-1.39	0.505	1.08	0.77-1.50	0.666
log(CXCL9)	1.09	0.92-1.31	0.317	0.97	0.76-1.22	0.781
log(IL17)	1.14	0.72-1.81	0.575	1.09	0.58-2.05	0.787
**log(MMP3)**	**1.51**	**1.08-2.12**	**0.017**	**1.73**	**1.14-2.64**	**0.011**
log(opn)	1.45	0.86-2.45	0.163	1.55	0.77-3.11	0.216
log(sbaff)	1.14	0.72-1.80	0.571	0.75	0.33-1.71	0.494
**log(ST2)**	**2.01**	**1.34-3.01**	**<0.001**	**2.77**	**1.59-4.84**	**<0.001**
log(B7H3)	1.13	0.73-1.77	0.579	1.54	0.98-2.44	0.062
log(DKK3)	2.1	0.91-4.86	0.084	1.54	0.83-2.84	0.171
log(IL1RACP)	1.09	0.48-2.48	0.834	1.39	0.49-3.92	0.536
log(CSF1)	1.02	0.85-1.23	0.798	0.98	0.78-1.23	0.856
log(CCL5)	0.83	0.67-1.03	0.097	0.71	0.58-0.88	0.002

Table S12. Multivariate analyses for correlation of cGVHD biomarkers and NRM in BMTCTN 0201 cohort

PB				BM			
**Multivariate Biomarker Analysis**							
**Variable**	**HR**	**95% CI**	**p-value**	**Variable**	**HR**	**95% CI**	**p-value**
**log(ST2)**	**2.01**	**1.34-3.01**	**<0.001**	**log(ST2)**	**2.46**	**1.42-4.29**	**0.001**
				**log(CD163)**	**3.31**	**1.22-9.02**	**0.019**
**Multivariate + clinical covariates** (conditioning regimen in PB and HLA mismatch in BM)							
**Variable**	**HR**	**95% CI**	**p-value**	**Variable**	**HR**	**95% CI**	**p-value**
**log(ST2)**	**2.09**	**1.38-3.19**	**<0.001**	**log(ST2)**	**2.31**	**1.28-4.17**	**0.005**
				**log(CD163)**	**3.09**	**1.15-8.33**	**0.026**

Table S13. Univariate analyses for correlation of cGVHD biomarkers and NRM in BMTCTN 1202 cohort

	PB			BM		
**Variable**	**HR**	**95% CI**	**p-value**	**HR**	**95% CI**	**p-value**
**log(CD163)**	2.05	0.94-4.48	0.071	**28.52**	**1.63-499**	**0.022**
log(CXCL10)	1.02	0.90-1.17	0.714	1.07	0.76-1.51	0.698
log(CXCL9)	1.02	0.90-1.17	0.729	1.14	0.82-1.59	0.434
log(IL17)	1.00	0.91-1.09	0.926	0.79	0.62-1.01	0.063
**log(MMP3)**	**1.31**	**1.06-1.61**	**0.011**	1.40	0.92-2.15	0.117
**log(ST2)**	**1.88**	**1.44-2.45**	**<0.001**	**2.44**	**1.17-5.07**	**0.017**
**log(DKK3)**	**1.92**	**1.21-3.06**	**0.006**	**3.47**	**1.46-8.26**	**0.005**

Table S14. Multivariate analyses for correlation of cGVHD biomarkers and NRM in BMTCTN 1202 cohort

PB				BM			
**Multivariate Biomarker Analysis**							
**Variable**	**HR**	**95% CI**	**p-value**	**Variable**	**HR**	**95% CI**	**p-value**
**log(ST2)**	**1.88**	**1.44-2.45**	**<0.001**	**log(DKK3)**	**3.40**	**1.34-8.62**	**0.004**
				**Log(CD163)**	**21.77**	**1.26-375**	**0.025**
**Multivariate + clinical covariates** (age and donor type in PB and none in BM)							
**Variable**	**HR**	**95% CI**	**p-value**	**Variable**	**HR**	**95% CI**	**p-value**
**log(ST2)**	**1.63**	**1.30-2.04**	**<0.001**				

Table S15. Details of samples' collection and processing BMTCTN 0201 cohort

551 participants from 45 locations were enrolled. Patient sample aliquot processing was performed on the day of collection by the clinical sites and stored frozen at each participating site. Frozen patient sample aliquots were periodically batch-shipped to the NHLBI Biorepository. NHLBI received 10,075 total vials from participating centers for this protocol and based on BMTCTN records, there were 40 vials received at NHLBI that had to be destroyed due to being broken or being linked to discrepant data.

The collection's schedule from the 0201 proposal is shown below:

Subject	Sample Type	Sample Aliquot Volume & Quantity	Pre-HSC Donation Baseline	Pre-Conditioning Baseline	Post-Transplant				
					Day 90	Day 180	Day 330	Day 365	Day 730
Patient	Serum	0.25-1.0 mL 3-10 aliquots		X		X	X	X	X
	Plasma (EDTA)	0.25-1.0 mL 2-6 aliquots			X	X		X	X
Donor	Serum	1.0 mL Single aliquot for majority of donors	X						

Note 1: Patient sample aliquot processing was performed con the day of collection by the clinical sites. See Appendix C of protocol for a description of peripheral blood processing procedures. Frozen patient sample aliquots were periodically batch-shipped to the NHLBI Biorepository. All samples being stored at -80ºC.

Note 2: Donor sample aliquot processing was performed next day by a 0201 project laboratory. See Appendix C of protocol for a description of peripheral blood processing procedures. Frozen donor sample aliquots were batch-shipped at the end of the study to the BMT CTN Biorepository. All samples being stored at -80ºC.

Table S16. Details of samples' collection and processing BMTCTN 1202 cohort

1860 participants from 42 locations were enrolled. Samples were ultimately collected from 1709 patients. All samples were sent priority overnight to the BMTCTN Biorepository and processed and stored at -80ºC for future research. 15,201 blood collection tubes were received at the biorepository from participating sites for processing; 15,035 arrived in a condition that allow for processing and storage.

The collection's schedule from the 1202 proposal is shown below:

Biomarker Approach	Sample Type	Subjects	Pre-HCT Day -1 or 0	Days Post-HCT						
				7 ± 2	14 ± 2	21 ± 2	28 ± 2	42 ± 3	56 ± 3	90 ±10
Proteomic	Serum (5 mL blood)	All Patients[1]	X	X	X	X	X	X	X	X
	EDTA Plasma (5 mL blood)		X	X	X	X	X	X	X	X
Gene Expression	PAXgene Lysates-stabilized whole blood RNA (15 mL blood)	240 Patients[2]				X			**X**	X
	CytoChex tube for Immunophenotyping (5 mL blood)	240 Patients[2]				X			**X**	X

Notes:

[1] Subjects weighing between 10 and 20 kg will only be required to provide a 5 mL blood sample (2.5 mL into a red-top tube for serum and 2.5 mL into an EDTA tube for plasma) for proteomic studies.

[2] This is a subset of the enrolled patients and will require additional samples to be collected at Days 21, 56 and 90 for gene expression studies. All proteomic samples will be collected per protocol for these patients.

Table S17: Details of ELISAs used for candidate protein testing

Protein name	Description	Commercial ELISA provider	Plasma dilution	LLOD	ULOD
CCL15	C-C motif chemokine ligand 15	R&D Duoset	1:25	15 pg/ml	1000 pg/ml
CD163	CD163 molecule	R&D Quantikine	1:10	1.5 ng/ml	100 ng/ml
CXCL10	C-X-C motif chemokine ligand 10	R&D Quantikine	1:20	8 pg/ml	1000 pg/ml
CXCL9	C-X-C motif chemokine ligand 9	RayBiotech	1:20	8 pg/ml	6000 pg/ml
IL17	interleukin 17	R&D Quantikine	undiluted	0.3 pg/ml	15 pg/ml
MMP3	matrix metallopeptidase 3	R&D Duoset	1:25	31 pg/ml	2000 pg/ml
OPN (SPP1)	Osteopontin, secreted phosphoprotein 1	R&D Duoset	1:200	62 pg/ml	4000 pg/ml
sBAFF (TNFSF13B)	B-cell activating factor	R&D Quantikine	1:10	62 pg/ml	4000 pg/ml
ST2	Stimulation-2, IL-33 receptor, Interleukin-1 receptor-like 1	R&D Quantikine	1:50	31 pg/ml	2000 pg/ml
B7H3 (CD276)	CD276 molecule	R&D Quantikine	undiluted	0.3 ng/ml	100 ng/ml
DKK3	dickkopf WNT signaling pathway inhibitor 3	R&D Duoset	1:50	18 pg/ml	2500 pg/ml
IL1RACP	interleukin 1 receptor accessory protein	R&D Duoset	1:200	31 pg/ml	2000 pg/ml
CSF1 (MCSF)	colony stimulating factor 1, Macrophage colony stimulating factor	R&D Duoset	undiluted	15 pg/ml	1000 pg/ml
CCL5	C-C motif chemokine ligand 5	R&D Duoset	1:100	15 pg/ml	1000 pg/ml
DCD	dermcidin	Abbexa	undiluted	0.8 ng/ml	25 ng/ml
DSG1	desmoglein 1	Abbexa	undiluted	0.4 ng/ml	12.5 ng/ml
CHI3L1 (YKL-40)	chitinase 3 like 1	R&D Quantikine	1:50	62 pg/ml	4000 pg/ml
FGL2	fibrinogen like 2	Biolegend	1:5	0.5 ng/ml	32 ng/ml

Supplemental Material references

1. Vander Lugt MT, Braun TM, Hanash S, et al. ST2 as a marker for risk of therapy-resistant graft-versus-host disease and death. N Engl J Med 2013;369:529-39.
2. Paczesny S, Krijanovski OI, Braun TM, et al. A biomarker panel for acute graft-versus-host disease. Blood 2009;113:273-8.
3. Fiema B, Harris AC, Gomez A, et al. High throughput sequential ELISA for validation of biomarkers of acute graft-versus-host disease. Journal of visualized experiments : JoVE 2012.
